# Phytochemical Analysis and Evaluation of Antioxidant, Antidiabetic, and Anti-inflammatory Properties of Aegle marmelos and Its Validation in an In-Vitro Cell Model

**DOI:** 10.7759/cureus.70491

**Published:** 2024-09-30

**Authors:** Saravanakumar Venkatesan, Anusha Rajagopal, Balasubramanyam Muthuswamy, Viswanathan Mohan, Nagaraj Manickam

**Affiliations:** 1 Department of Vascular Biology, Madras Diabetes Research Foundation; Affiliated to University of Madras, Chennai, IND; 2 Department of Cell and Molecular Biology, Madras Diabetes Research Foundation; Affiliated to University of Madras, Chennai, IND; 3 Department of diabetology, Madras Diabetes Research Foundation; Dr. Mohan's Diabetes Specialities Centre, Chennai, IND

**Keywords:** aegle marmelos, anti-inflammatory, antioxidant, hyperglycemia, phyto pharmacology

## Abstract

Introduction: Persistent hyperglycemia significantly increases oxidative stress and inflammation resulting in multiple cellular and molecular alterations which further exacerbate the diabetes associated complications. *Aegle marmelos (L.) Corrêa *is a medicinal plant used in the Indian system of medicine for treating various disorders including diabetes. However, studies on phytoconstituents and their pharmacological activity of this plant are limited. Therefore, we aimed to determine the phytochemical components, evaluate the antidiabetic activity, anti-inflammatory activity, and antioxidant activity of *A. marmelos* leaf extract, and validate its mechanistic effects in an in vitro cell model.

Methods: The qualitative and quantitative analysis of the different phytoconstituents in the extract was determined using standardized protocols. The antioxidant activity of the extract was evaluated by 2,2-di-phenyl-1-picrylhydrazyl (DPPH) radical scavenging capacity assay and ferric reducing antioxidant power (FRAP) assay. The antidiabetic activity of the extract was evaluated by α-amylase inhibition and α-glucosidase inhibition assay. The anti-inflammatory activity was studied using an albumin denaturation assay. In addition, the pharmacological effect(s) of leaf extract was checked in the normal rat kidney fibroblast cells (NRK-49F) under high glucose conditions. Intracellular reactive oxygen species (ROS) generation was measured by fluorometry using fluorescence probe 2′,7′-dichlorodihydrofluorescin diacetate (DCF-DA). mRNA expression of inflammatory markers including inducible nitric oxide synthase (iNOS) and tumor necrosis factor-alpha (TNF-α) was studied using real-time quantitative polymerase chain reaction (RT-qPCR). Cell migration was studied using cell scratch assay. Statistical analysis was performed using GraphPad Prism version 8.0.

Results: The phytochemical analysis of *A. marmelos *leaf extract revealed the presence of alkaloids, phenols, flavonoids, and saponins. The extract showed higher antioxidant activity in the DPPH (IC_50_=258.21 µg/mL) and FRAP assay (IC_50_=293.83 µg/mL). The extract exhibited prominent antidiabetic activity by inhibiting enzymes α-Amylase (IC_50_=73.2 µg/mL) and α-glucosidase (IC_50_=43.9 µg/mL). In addition, the extract showed effective anti-inflammatory activity by significantly inhibiting the denaturation of egg albumin (IC_50_=102.8 µg/mL). Further, the leaf extract significantly decreased the high glucose-induced ROS generation as well as inflammatory markers in rat fibroblast cell lines in a dose-dependent manner. Additionally, high glucose-induced cell migration as the measure of cell injury was effectively reduced by the extract treatment.

Conclusion: *A. marmelos *leaf* *extract was quantified to possess a substantial amount of important phytoconstituents that have promising pharmacological properties. Besides showing antidiabetic activity, the extract significantly combats the high glucose-induced ROS generation, inflammatory markers expressions, and cell migration. Further, in-depth studies and clinical trials are warranted so as to position these traditional remedies for the treatment of metabolic disorders.

## Introduction

In recent decades, plant-based medicine has gained recognition primarily because of its effectiveness, and ability to target numerous signaling pathways and have fewer side effects. Based on World Health Organization statistics, approximately 80% of people worldwide use traditional medicine to treat various disorders [1]. The therapeutic applications of plants were documented in ancient medical texts and are in practice worldwide. Plant-based medicine has been used in the Indian system of medicine, Traditional Chinese medicine, African countries, and Central American countries for treating various diseases including diabetes mellitus, hypertension, cardiovascular diseases, and infectious diseases. Almost 40% of pharmaceutical products are derived from natural products and important drugs like aspirin and artemisinin are developed from natural products. Metformin is the primary oral antidiabetic drug used to reduce blood glucose levels in type 2 diabetes mellitus patients. It is extracted from the plant Galega officinalis and has been traditionally used in Europe [2]. Recently, phlorizin, which was also originally extracted from the roots and bark of apple trees, has been shown to inhibit sodium glucose co-transporter-2 (SGLT2) activity and is used for the treatment of diabetes [3]. Oxidative stress plays a vital role in the development and progression of diabetes and its vascular complications. The disparity between reactive-oxygen species (ROS) generation and antioxidant defense mechanisms disrupts normal physiological function and causes detrimental effects on cellular structure and function. In diabetes, persistent hyperglycemia leads to increased ROS generation and inflammation, consequently worsening the cellular function by increasing insulin resistance, endothelial dysfunction, and inflammation by activating multiple signaling cascades [4]. Although plant-based medicine has been mentioned in ancient medicinal textbooks and used in the traditional system of medicine around the globe for thousands of years, there is a lack of studies on their quantitative determination of phytochemical constituents as well as their mechanistic mode of therapeutic actions [5]. This warrants continuous research and in-depth studies of natural products.

*Aegle marmelos* (L.) Corrêa, a deciduous tree belonging to the family Rutaceae is widely distributed throughout the Southeast Asian countries. It is a well-known medicinal plant used in the Indian system of medicine such as Siddha and Ayurveda for the treatment of diabetes, cardiovascular diseases, and other metabolic disorders, and its references date back to Vedic times [6]. The therapeutic properties of this tree have been recorded in well-known ancient medicinal literature Charaka Samhita [7]. It has been used as a medicine, food product, and nutritional supplement in the Indian subcontinent. All the parts of the plants such as leaves, fruits, roots, and leaves contain medicinal properties and are used for treating various complications [7,8]. Among different parts, leaves, and fruit were used predominantly in the drug formulations for treating diabetes, inflammation, microbial infection, gastric ulcers, and respiratory problems. While the phytochemical components and pharmacological activity of plant extract were limited, there is also a lack of in vitro cell-based mechanistic studies Therefore, this study aimed to determine the phytochemical composition and study the pharmacological properties such as antioxidant, anti-inflammatory, and antidiabetic activities (Type 2 diabetes) of *A. marmelos* leaf extract. We also validated their pharmacological effect on high glucose-induced ROS generation, cell migration, and inflammation in the kidney fibroblast cell line.

## Materials and methods

Plant collection and extract preparation

The leaves of *A. marmelos* were collected from Kancheepuram district, Tamil Nadu, India. The taxonomy of the plant was identified and authenticated by botanist. A voucher specimen (VB001) of the sample was deposited at the Madras Diabetes Research Foundation, India. The fresh leaves were collected and shade-dried for 3 days and pulverized to obtain a fine powder form and stored in airtight glass containers. The dry powder was then cold percolated with 70% ethanol for 3 days with occasional stirring and filtered using Whatman filter paper (Grade 1). It was followed by the concentration of the filtrate under reduced pressure using a rotary evaporator at 45°C. The concentrated extract was freeze-dried and stored at -20°C in an amber bottle for further experimental use.

Cell culture

Normal rat kidney fibroblast cell line (NRK-49F) was procured from ATCC (Manassas, VA, USA). It was cultured in Dulbecco’s Modified Eagle Medium (DMEM) supplemented with 10% fetal bovine serum (FBS) and 1% penicillin-streptomycin solution and maintained in an atmosphere of 5% CO2 at 37°C. To study the effect of leaf extract, cells were seeded in a multi-well plate with 50-60% confluence, serum starved for 24 hours, and treated with different doses of the leaf extract (50-500 µg/mL) followed by high glucose treatment (25 mM) for 48 hrs.

Qualitative analysis

The extract was subjected to qualitative analysis using standardized protocols for the identification of the main classes of phytochemicals such as alkaloids, saponin, terpenoids, cardiac glycosides, flavonoids, tannins, carotenoids, phenols, anthraquinones, and steroids [9].

Quantitative analysis

Determination of Total Flavonoid Content

The total flavonoid content in the extract was quantified by the aluminum chloride colorimetric method [10]. Briefly, to the extract aluminum trichloride (10%) and sodium nitrite (5%) were added, and after 10 min sodium hydroxide (0.001 M) was added and incubated for 25 min. Then, we measured the absorbance at 510 nm and the amount of flavonoids was calculated using the linear equation obtained from the calibration curve of quercetin as standard. The quantity of flavonoids was expressed in terms of quercetin equivalent mg/g extract.

Determination of Total Phenolic Content

The total phenolic content was quantified based on Folin-Ciocalteu’s method. Briefly, the extract was added to the Folin-Ciocalteu’s reagent (10%) and sodium carbonate (7%) and incubated at 25°C for 30 min. Then, the absorbance was measured at 760 nm. The phenolic content was determined using the linear equation obtained from the calibration curve of gallic acid as standard. The quantity of total phenol was expressed in terms of gallic acid equivalent in mg/g extract [10].

Determination of Total Alkaloid Content

Total alkaloid content was determined by dissolving the extract in 2N HCl and filtered using Whatman filter paper. Then it was washed with chloroform four times using a separating funnel and pH 7.0 was adjusted using sodium hydroxide (0.1 N). Bromocresol green solution (0.1 M) and phosphate buffer (0.4 M, pH 4.7) were added to the filtrate, incubated for 20 min, and made up to 10 mL using chloroform. The reaction mixture was stirred vigorously followed by the separation of the chloroform layer and the absorbance was measured at 470 nm. The total alkaloids were quantified from an atropine standard curve and expressed as atropine equivalent per mg/g extract [11].

Antioxidant assay

2,2-Diphenyl-1-Picrylhydrazyl (DPPH) Radical Scavenging Activity

The antioxidant activity of the extract was determined by the DDPH (2,2-Diphenyl-1-picrylhydrazyl) free radical scavenging assay [12]. Briefly, different concentrations of the extract were prepared (100-500 µg/mL) and mixed with the DPPH solution (0.1 mM) and made up with 4 mL of methanol. The reaction mixture was incubated at room temperature for 30 min in the dark. The control solution was obtained by adding methanol to the DPPH radical solution. The absorbance was measured at 517 nm. The results were determined by comparing the absorbance of the extract using ascorbic acid as standard. The scavenging activity percentage was calculated using the below formula:

% Antioxidant activity = ((control absorbance - sample absorbance)/(control absorbance)) X 100

Ferric Reducing Antioxidant Power (FRAP) Assay

Different concentration of the extract (100-500 µg/mL) was mixed with the phosphate buffer (0.2 M, pH=6.6) and ferric tripyridyl triazine (1%) and incubated at 50°C for 30 min. Then, 10% of trichloroacetic acid was added and centrifuged to remove insoluble particles. Finally, ferric chloride (1%) was added to the supernatant and the absorbance was measured at 700 nm [12]. Ascorbic acid was used as standard. The percentage of inhibition was calculated using the below formula:

% Antioxidant activity = ((control absorbance - sample absorbance)/(control absorbance)) X 100

Antidiabetic activity

α-Amylase Inhibitory Activity

Different concentration of the extract (50-250 µg/mL) or acarbose was added to the sodium phosphate buffer (20 mM, pH=6.9) containing enzyme α-amylase (2 U/mL) and incubated for 20 min at 37°C. Then, the substrate starch (1%) was added and incubated for 30 min at 37°C. After adding the dinitrosalicylic acid reagent (96 mM), the reaction mixture was placed in a boiling water bath for 15 min and cooled down at room temperature. Finally, the absorbance was measured at 540 nm [13]. The α-amylase inhibitory activity was determined using the following formula and the half-maximal inhibitory concentration (IC50) was determined from the graph.

% α-amylase inhibitory activity = ((control absorbance - sample absorbance)/(control absorbance)) X 100

α-Glucosidase Inhibitory Activity

Different concentration of the extract (50 - 250 µg/mL) or acarbose was added to the phosphate buffer (0.1 M, pH=6.9) and incubated with the enzyme α-glucosidase (1 U/mL) at 25°C for 15 min. After the incubation, p-nitrophenyl-α-D-glucopyranoside (5 mM) was added as a substrate to the reaction mixture and incubated at 37°C for 20 min. The reaction was stopped by adding sodium bicarbonate (0.1 M). Then, the absorbance was measured at 405 nm [13]. The α-glucosidase inhibitory activity was determined using the following formula and IC50 was estimated from the graph.

% α-Glucosidase inhibitory activity = ((control absorbance - sample absorbance)/(control absorbance)) X 100

Anti-inflammatory assay

The anti-inflammatory activity of the extract was analyzed using a heat-induced egg albumin denaturation assay. Briefly, different concentration of the extract was prepared (50-250 µg/mL) and mixed with the BSA (1%) and phosphate-buffered saline (PBS) followed by incubation at 37°C for 20 min. Then, the temperature was increased to 70°C for 10 min and absorbance was measured at 660 nm [12]. Diclofenac sodium was used as a standard. The anti-inflammatory inhibition percentage was calculated using the following formula:

% Anti-inflammatory activity = ((control absorbance - sample absorbance)/(control absorbance)) X 100

Cell viability assay

The effect of the extract on cell viability was studied using 3-(4,5-dimethyl-2-thiazolyl)-2,5-diphenyl-2H-tetrazolium bromide (MTT) assay in NRK-49F cells. Cells were seeded in a 96-well plate at the seeding density of 2000 cells/well and treated with different concentrations of the extract (100 µg/mL - 5000 µg/mL) for 48 hrs. After treatment, the medium was changed and MTT solution (0.5 mg/mL) was added and incubated for 4 hrs at 37°C. After incubation, the medium was removed, followed by the addition of dimethyl sulfoxide (DMSO) to solubilize the formazan crystals. Finally, absorbance was measured using a multimode plate reader at 540 nm [13]. The percentage of cell viability was calculated using the following formula:

% Cell viability = (Treated absorbance)/(control absorbance) X 100

Determination of intracellular ROS generation

The effect of the extract on high glucose-induced intracellular ROS generation was studied using fluorescent probe 2′,7′-dichlorodihydrofluorescin diacetate (DCF-DA). Cells were seeded in a 12-well plate and the serum was starved overnight. After serum starvation, cells were treated with different concentrations of the extract and high glucose (25 mM) for 48 hrs. At the end of the incubation period, cells were washed with PBS, and DCF-DA (10 μM) was added and incubated for 30 min at 37°C. The levels of intracellular ROS were measured using a multimode plate reader at excitation and emission wavelengths of 488 nm and 520 nm, respectively [14]. The results were represented as relative fluorescence units (RFU) (%).

Real-time quantitative polymerase chain reaction (RT-qPCR)

Total RNA was isolated from the cells using the TRIzol reagent (Takara Bio, Kusatsu, Japan). 500 ng of RNA was reverse transcribed to complementary DNA using a PrimeScript cDNA synthesis kit (Takara Bio, Kusatsu, Japan). The cDNA was used for real-time PCR analysis of target iNOS and TNF-α using SYBR Green (Takara, Kusatsu, Japan) in Light Cycler 96 Analyser system (Roche, Basel, Switzerland) under appropriate cycle conditions. ΔΔct method was used to determine the fold expression by normalizing to endogenous glyceraldehyde-3-phosphate dehydrogenase (GAPDH). The primers used are stated in Table 1.

**Table 1:** List of primers used in this study iNOS: inducible nitric oxide synthase; TNF-α: tumor necrosis factor-alpha; GAPDH: glyceraldehyde 3-phosphate dehydrogenase

Gene	Forward Primer	Reverse Primer
iNOS	TGGTGAGGGGACTGGACTTT	TGTTGGGCTGGGAATAGCAC
TNF-α	ATGGGCTCCCTCTCATCAGT	GCTTGGTGGTTTGCTACGAC
GAPDH	AACCCATCACCATCTTCCAG	GCCATCCACAGTCTTCTGAG

Cell migration assay

Cells were seeded in a six-well plate, and the scratch was made in the cell monolayer with a pipette tip. After creating a scratch, cells were washed with PBS to remove the cell debris followed by the addition of fresh medium. The scratch area was photographed using an inverted microscope. Then, cells were treated with different concentrations of the extract (100 - 500 µg/mL) followed by high glucose treatment (25 mM) and incubated for 16 hrs. At the end of the experiment, the images of the scratch area were captured. The cell migrated area was analyzed by comparing the scratch area at two different time points [14].

Statistical analysis

Statistical analysis was performed using GraphPad Prism version 8.0 (GraphPad Software, CA, USA). One-way analysis of variance (ANOVA) was used to analyze the difference between the groups followed by the post-hoc analysis using Tukey multiple comparison test. All the experiments were performed for at least three independent times. The difference between the groups with p<0.05 were considered statistically significant. Results are presented as the mean ± standard error of the mean (SEM).

## Results

Phytochemical analysis

The freeze-dried *A. marmelos* leaf extract was prepared from dried leaf powder and the yield was found to be 9.54±0.20%. To study the presence of phytochemical components, the extract was subjected to preliminary phytochemical analysis which revealed the presence of phytochemicals such as tannins, saponins, flavonoids, alkaloids, terpenoids, cardiac glycosides, and phenols whereas carotenoids, steroids, phlobatannins, and anthraquinones were absent (Table 2). Then, we quantitatively estimated the medicinally important phytochemicals such as alkaloids, phenols, and flavonoids in the extract. “The quantity of alkaloids, flavonoids, and total phenols present in the extract are listed in Table 3”.

**Table 2:** Phytochemical constituents of Aegle marmelos leaf extract + : detected; - : not detected

S. No.	Phytochemical	Result
1	Alkaloids	+
2	Saponins	+
3	Terpenoids	+
4	Cardiac glycosides	+
5	Flavonoids	+
6	Tannins	+
7	Phenols	+
8	Carotenoids	-
9	Phlobatannins	-
10	Anthraquinones	-
11	Steroids	-

**Table 3:** Quantitative analysis of Aegle marmelos leaf extract

S. No.	Phytochemical Component	Concentration
1	Total alkaloid content (mg atropine/g extract)	18.34±1.60
2	Total phenolic content (mg gallic acid/g extract)	25.51±1.11
3	Total flavonoid content (mg quercetin/g extract)	27.95±0.50

Antioxidant activity

DPPH Antioxidant Activity

**Figure 1:**
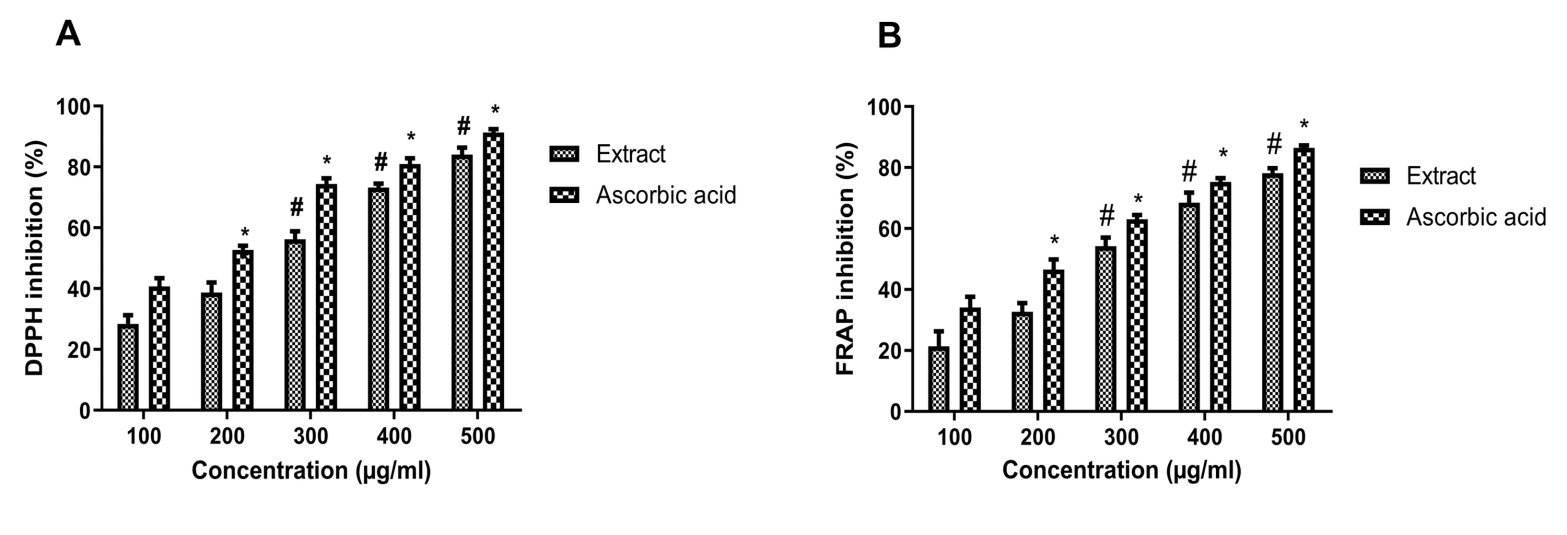
The antioxidant activity of the extract In both antioxidant activity assays (DPPH (A) and FRAP (B) assays), the extract showed dose-dependent inhibition and results were compared with the standard drug, ascorbic acid. Data were represented as mean ± SEM (n=3). *p<0.05 compared with standard drug (100 µg/mL), #p<0.05 compared with extract (100 µg/mL). DPPH: 2,2-di-phenyl-1-picrylhydrazyl, FRAP: ferric reducing antioxidant power

The antioxidant activity of the extract was studied using DPPH assay and the bar graph revealed that the antioxidant activity was increased significantly in a dose-dependent manner and the maximum inhibition was found at 500 µg/mL (Figure 1A). At higher concentrations, the extract showed high antioxidant properties compared to that of reference standard ascorbic acid. The IC_50_ value of extract and ascorbic acid was found to be 258.21 µg/mL and 161.31 µg/mL, respectively.

Ferric Reducing Antioxidant Power (FRAP) Assay

The FRAP inhibitory activity of the extract was studied and compared with ascorbic acid. Similar to the DPPH assay, the antioxidant activity of the extract was increased in a dose-dependent manner and the maximum inhibition was found at 500 µg/mL (Figure 1B). At the highest concentration of 500 µg/mL, the standard compound exhibited 86.2% inhibition and the extract showed 78.1% inhibition. The IC_50_ value of the extract and ascorbic acid were 293.83 µg/mL and 218.04 µg/mL, respectively.

Antidiabetic activity

α-Amylase Inhibitory Activity

**Figure 2:**
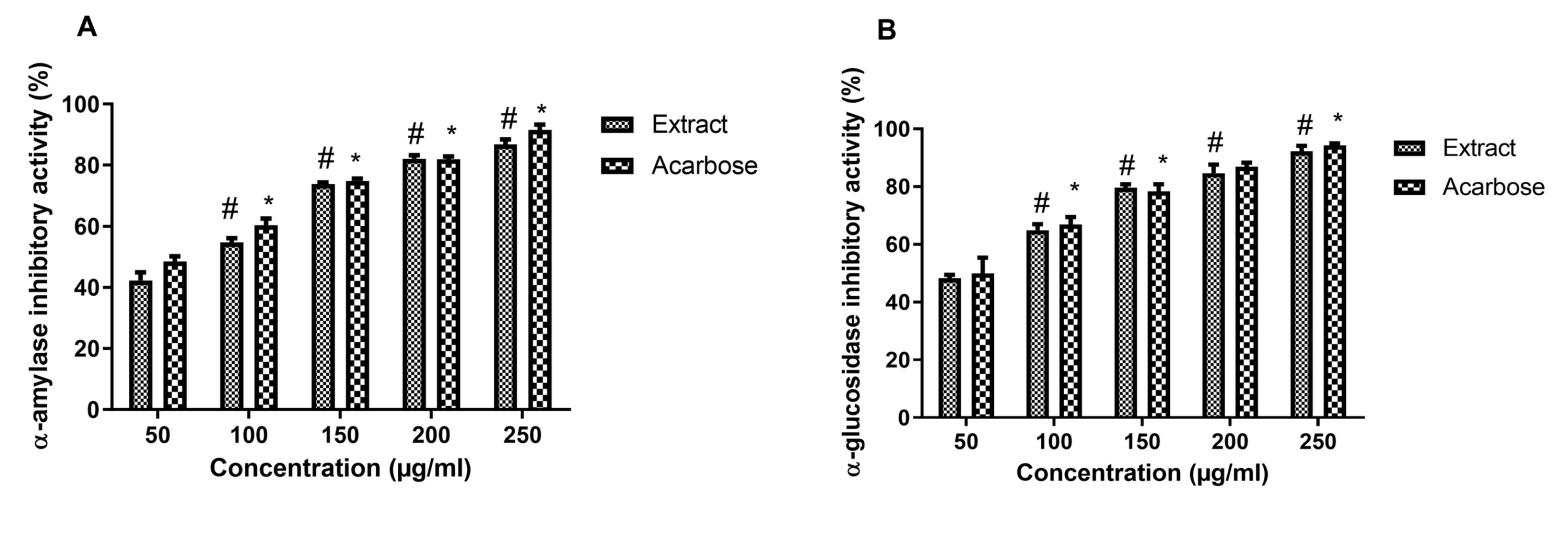
The antidiabetic activity of the extract In both antidiabetic assays (α-amylase inhibition assay (A) and α-glucosidase inhibition assay (B)), the extract showed dose-dependent inhibition of both enzymes, and results were compared with the standard drug acarbose. Data were represented as mean ± SEM (n=3). *p<0.05 compared with standard drug (50 µg/mL), #p<0.05 compared with extract (50 µg/mL).

The antidiabetic potential of the extract was evaluated using α-amylase inhibitory activity. The results revealed that α-amylase inhibitory activity of the extract was significantly increased dose-dependently and the maximum inhibition was found at 250 µg/mL (Figure 2A). At the highest concentration of 250 µg/mL, the standard compound exhibited 92% inhibition and the extract showed 86.1% inhibition. The inhibitory effect of the extract on α-amylase was comparable with the reference standard acarbose. The IC_50_ value of the extract was found to be 73.2 µg/mL. The standard positive control acarbose showed an IC_50_ value of 50.9 μg/mL.

α-Glucosidase Inhibitory Activity

We further confirmed the antidiabetic activity of the extract using α-glucosidase inhibitory assay. Similarly, the extract significantly inhibited the α-glucosidase activity in a dose-dependent manner, and the maximum inhibition was found at 250 μg/mL (Figure 2B). At the highest concentration of 250 µg/mL, the standard compound exhibited a 94.1% inhibition and the extract showed inhibition of 91.5%. The IC_50_ value of the extract was found to be 43.9 µg/mL. The standard positive control acarbose exhibited an IC_50_ value of 33.58 μg/mL.

Anti-inflammatory activity

**Figure 3:**
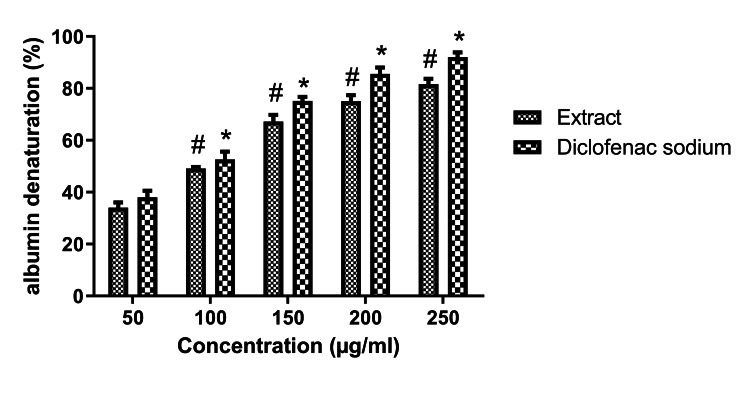
The anti-inflammatory activity of the extract The extract showed significant dose-dependent denaturation of protein albumin and results were compared with diclofenac sodium. Data were represented as mean ± SEM (n=3).*p<0.05 compared with standard drug (50 µg/mL), #p<0.05 compared with extract (50 µg/mL).

The anti-inflammatory activity of the extract was studied using the denaturation of egg albumin method. The inhibitory activity of the extract was increased significantly (Figure 3) in a dose-dependent manner. At the highest concentration of 250 µg/mL, the standard compound exhibited 92% inhibition and the extract showed 81% inhibition. The IC_50_ value of extract and diclofenac sodium was 102.8 μg/mL and 84.3 μg/mL, respectively.

Effect of the extract on intracellular reactive-oxygen species levels

**Figure 4:**
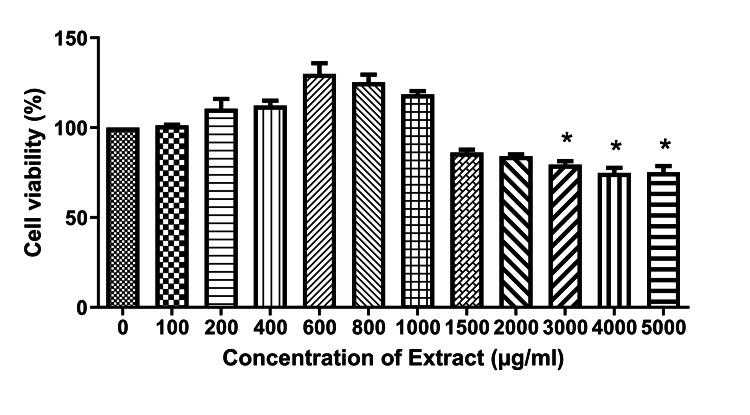
The effect of the extract on cell viability in NRK-49F cell line The extract did not significantly reduce the cell viability till 2000 µg/mL. Data were represented as mean ± SEM (n=3). *p<0.05 compared to without treatment condition.

We next aimed to confirm the efficiency of the extract on the cell line by investigating its effect on cell viability through the MTT assay. The results showed that the extract did not significantly reduce the cell viability up to 2.0 mg/mL when compared with the control (Figure 4).

**Figure 5:**
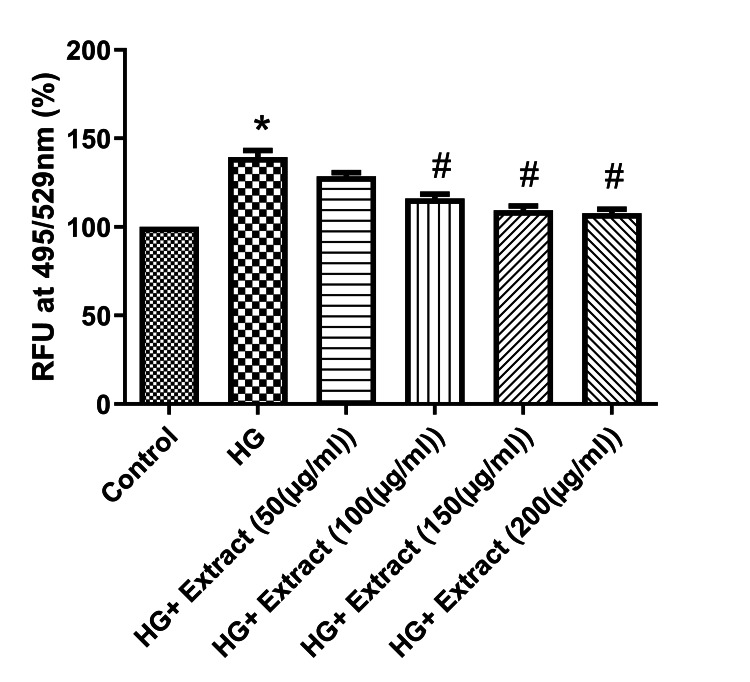
The effect of the extract on high glucose-induced ROS generation in NRK-49F cell line The extract treatment significantly decreased the ROS generation under high glucose in a dose-dependent manner. Data were represented as mean ± SEM (n=3). *p<0.05 compared to control; #p<0.05 compared to HG. HG: high glucose; RFU: relative fluorescence units

Then, we validated the antioxidant effect of the extract on combating high glucose-induced ROS generation in NRK-49F cells. The increased levels of ROS generation are an indicator of oxidative stress upon high glucose treatment. High glucose treatment significantly increased the ROS generation by up to 50% when compared with the control (Figure 5). The extract treatment significantly decreased the high glucose-mediated ROS generation dose-dependently at the concentration ranging from 100 to 200 μg/mL.

Effect of the extract on inflammatory markers

**Figure 6:**
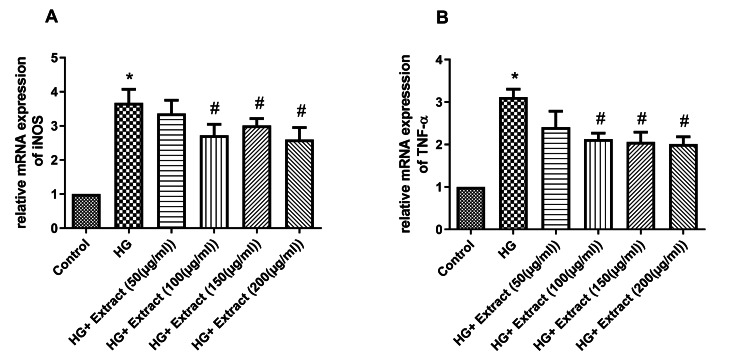
The effect of the extract on inflammatory markers in NRK-49F cell line Extract treatment decreased the inflammatory markers iNOS (A) and TNF-α (B) in a dose-dependent manner under high glucose conditions. Data were represented as mean ± SEM (n=3). *p<0.05 compared to control; #p<0.05 compared to HG. HG: high glucose; iNOS: inducible nitric oxide synthase; TNF-α: tumor necrosis factor alpha

We have also validated the anti-inflammatory effect of the extract under high glucose conditions in NRK-49F cells. The inflammatory markers including iNOS and TNF-α were increased significantly under the high glucose condition (Figure 6). The extract treatment significantly decreased the mRNA expression of iNOS dose-dependently at the concentration ranging from 100 µg/mL to 200 µg/mL. Also, the extract treatment significantly decreased the mRNA expression of TNF-α dose-dependently at concentrations ranging from 100 µg/mL to 200 µg/mL.

Effect of the extract on cell migration

**Figure 7:**
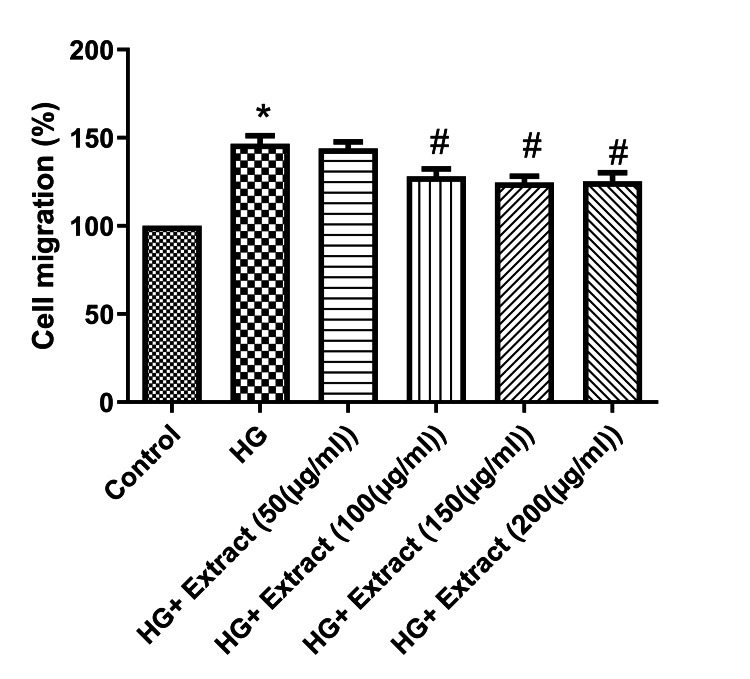
The effect of the extract on cell migration in NRK-49F cell line Extract treatment significantly decreased the cell migration when compared with the high glucose condition in a dose-dependent manner. Data were represented as mean ± SEM (n=3). *p<0.05 compared to control; #p<0.05 compared to HG. HG: high glucose

Next, we examined the effect of the extract on high glucose-induced cell injury by studying the cell migration under high glucose treatment in NRK-49F cells. The cell migration was significantly higher under high glucose conditions when compared with the control. Conversely, the extract treatment reduced the cell migration significantly in a dose-dependent manner under high glucose conditions (Figure 7).

## Discussion

Medicinal plants are the main sources of bioactive components with notable pharmacological activity, which play a prominent role in the prevention or treatment of different diseases due to their safety and therapeutic properties. Several medicinal plants have been employed in traditional medicine throughout the world for the treatment of diabetes and its related complications [2]. *A. marmelos* is a medicinal plant used in the Indian system of medicine for the treatment of various disorders including diabetes and cardiovascular disorders [6]. Though different parts of the plant such as leaf, bark, fruit, seed, and root have been traditionally used for potential medicinal benefits, studies on comprehensive profiling of phytoconstituents and pharmacological activity are very limited.

Our study revealed that different classes of phytoconstituents were present in the ethanolic extract of *A. marmelos* leaf extracts such as alkaloids, saponins, phenols, flavonoids, and tannins. Few other studies also demonstrated the presence of different classes of biochemical compounds in the leaf such as alkaloids, coumarins, essential oils, vitamins, carotenoids, and flavonoids [13,15]. We further quantified the alkaloids, total phenols, and flavonoids and found that alkaloid content was found to be higher in our extract, but the phenols and flavonoids were comparable with other studies [15]. A previous study confirmed the presence of quercetin, gallic acid, ferulic acid, and caffeic acid in the leaf extract which has beneficial medicinal properties [16]. These findings highlight the presence of a substantial amount of phytocomponents in *A. marmelos* leaf extract and support its potential therapeutic utility in disease management. Alkaloids account for almost 20% of secondary metabolites identified in plants. It has been studied that alkaloids derived from plants have anti-inflammatory properties by reducing the pro-inflammatory protein complexes associated with inflammatory signaling pathways and antioxidant properties by scavenging excessive ROS generation [17]. Flavonoids have been shown to regulate glucose metabolism, oxidative stress, and inflammation by reducing cytokine production, targeting transforming growth factor beta (TGF-β1)/Smad signaling and intracellular ROS levels [18].

Avula et al. identified several bioactive molecules such as aegeline, umbelliferone, marmesinin, angelicin, and marmelosine in different parts of *A. marmelos* [19]. Another study also reported the presence of prominent phytochemicals in the extract such as phytol, eugenol, rutin, and auraptene [6]. Phytol plays a promising role in the management of insulin resistance by modulating the transcription activity of peroxisome proliferator-activated receptor alpha and retinoid X receptor, increasing the serum adiponectin level and decreasing the level of TNF-α [20]. Eugenol has potential antidiabetic and anti-inflammatory properties by increasing glucose uptake by activating glucose transporter type 4 translocation and phosphorylation of adenosine monophosphate-activated protein kinase and decreasing lipid levels and oxidative damage in diabetes-induced animal models. In addition to the previously mentioned benefits, studies have demonstrated that eugenol and phytol scavenge free radical production in peripheral tissues and prevent the detrimental effects of free radicals [21].

From the previous studies, the activity of α-amylase and α-glucosidase was important for postprandial hyperglycemia. Inhibition of these enzymes lowers blood glucose and exhibits antidiabetic activity [22,23]. In our study, we found that extract potentially inhibits carbohydrate metabolizing enzymes such as α-amylase (IC_50_=73.2 µg/mL) and α-glucosidase (IC_50_=43.9 µg/mL). In a previous study, the antidiabetic activity of the leaf extract was studied using α-amylase (IC_50_=123.65 µg/mL) and α-glucosidase (IC_50_=141.6 µg/mL) inhibition assays [13] and comparatively, inhibition of the enzymes was lower than our study. In this study, results of in vitro antioxidant assays such as FRAP (IC_50_=293.83 μg/mL) and DPPH (IC_50_=258.21 μg/mL) showed the dose-dependent free radical scavenging potential of extract and IC_50_ values are in line with other studies [24,25]. The reduction capacity of the extract shows that it has potential polyphenols and flavonoids which have electron donor capacity and may reduce the oxidized metabolites in the circulation.

Our leaf extract showed no cytotoxicity up to a concentration of 2 mg/mL in rat kidney cells. Previous studies also confirmed that leaf extract does not reduce cell viability at lower concentrations and leaf extract (250 µg/mL) protects the cells against high glucose-mediated cell cytotoxicity [13,16]. Oxidative stress is known to play a prominent role in the pathogenesis of diabetes and its complications. A high glucose environment disrupts redox equilibrium, resulting in excessive ROS formation and activating a series of complicated signaling cascades, exacerbating the structural and functional abnormalities in renal cells. Our results also found that high glucose significantly increased the ROS level compared with the control. Few studies demonstrated the role of *A. marmelos* fruits in protecting cells from oxidative stress by increasing the circulatory antioxidant enzymes employing animal models [26,27]. Also, recently it was reported that *A. marmelos* leaf extract reduced the high glucose-mediated ROS generation in HepG2 cells [13,28]. Further, the leaf extract of *A. marmelos* increased the antioxidant levels of superoxide dismutase, catalase, and glutathione peroxidase and decreased the malondialdehyde in a diabetes-induced mouse model [16]. Similarly, our results showed the beneficial effect of the leaf extract in combating the high glucose-mediated excessive ROS generation in the kidney cells.

In diabetes induced animal model, the leaf extract reduced the circulatory levels of inflammatory markers such as Interleukin-1 beta (IL-1β), Interleukin 6 (IL-6), and TNF-α [16]. The roots and stem barks of *A. marmelos* also decreased the inflammatory enzymes (cyclooxygenase-1 and cyclooxygenase-5), and pro-inflammatory cytokines (IL-1β, macrophage inflammatory protein-1 alpha and IL-6) and increased the anti-inflammatory cytokine (Interleukin 2) in lipopolysaccharide treated macrophage cell line (RAW 264.7). The anti-inflammatory potential was further validated in an inflammation-induced mouse model by decreasing the thickness of paw edema [29]. In our study also, we demonstrated the protective effect of the extract on high glucose-driven inflammatory markers expression (iNOS and TNF-α) in the renal cells. Studies revealed that high glucose-driven ROS generation accelerates oxidative stress and has an impact on fibroblast cell migration, thus initiating myofibroblast activation. We found that leaf extract significantly reduced cell migration under high glucose conditions. Therefore, *A. marmelos* leaf extract plays a key role in restricting the migration of cells induced by high glucose conditions and could be further studied for delaying diabetes-mediated cell injury. Taken together, *A. marmelos* leaf extract holds great promise for the prevention and treatment of various diseases including diabetes, hypertension, and cardiovascular disease as it contains different classes of phytocomponents and medically important pharmacological properties.

Limitations

Though we have studied the phytoconstituents and biological activities of the *A. marmelos* leaf extract using an in vitro model, the exact mechanistic action of *A. marmelos* leaf extract was not explored in this study. Precise mechanistic action of extract in subjugating oxidative stress and inflammation under high glucose conditions will be addressed in our future studies. Further studies should be carried out to identify the active biomolecules that play a substantial role in the therapeutic action of the extract. Substantial in vivo studies are necessary for investigating the effective and safe dose, bioavailability, and pharmacokinetics of *A. marmelos* leaf extract.

## Conclusions

We have quantified and demonstrated that *A. marmelos* leaf extract contains different classes of phytochemicals and has significant amounts of important phytochemicals such as flavonoids, total phenols, and alkaloids. The extract exhibited potential antioxidant activity, antidiabetic activity, and anti-inflammatory activity which was evident from the pharmacological assays. The extract had no effect on cell viability at concentrations up to 2 mg/mL. This is the first study to validate the pharmacological impact of *A. marmelos* leaf extract in an in vitro model normal rat kidney cell. With a mechanistic mode of action approach, our study has demonstrated the antioxidant activity of *A. marmelos* leaf extract against high glucose-induced ROS generation. Similarly, the extract ameliorated the mRNA expression of inflammatory markers under high glucose conditions. Further, the extract substantially reduced the high glucose-mediated cell migration linked to cell injury. While our study is unique in endorsing the different pharmacological actions of *A. marmelos* leaf extract, further in-depth studies and clinical trials are warranted so as to position these traditional remedies for the treatment of metabolic disorders.
